# Axial length changes in highly myopic eyes and influence of myopic macular complications in Japanese adults

**DOI:** 10.1371/journal.pone.0180851

**Published:** 2017-07-07

**Authors:** Hideharu Ohsugi, Yasushi Ikuno, Tomohiro Shoujou, Kanako Oshima, Eiko Ohsugi, Hitoshi Tabuchi

**Affiliations:** 1 Department of Ophthalmology, Tsukazaki Hospital, Himeji, Japan; 2 Ikuno Eye Center, Osaka, Japan; Soochow University Medical College, CHINA

## Abstract

**Purpose:**

To investigate changes of the axial length in normal eyes and highly myopic eyes and influence of myopic macular complications in Japanese adults.

**Study design:**

Retrospective longitudinal case series.

**Methods:**

The changes in the axial length of 316 eyes from 316 patients (mean age, 63.8 ± 9.0 years; range, 34–82; 240 females) examined using IOLMaster with a follow-up period of at least 1 year were studied. This study included 85 non-highly myopic eyes (|refractive error| ≤ 5 diopters; 63 females; non-highly myopic group), 165 highly myopic eyes (refractive error ≤ −6 diopters or axial length ≥ 26 mm; 124 females) without macular complications (no complications group), 32 eyes (25 females) with myopic traction maculopathy (MTM group), and 34 eyes (28 females) with myopic choroidal neovascularization (CNV group).

**Results:**

All groups showed a significant increase in the axial length during the follow-up period (mean follow-up, 28.7 ± 16.8 months; range, 12–78) (P < 0.01). Changes in the axial length/year in the no complications group (0.041 ± 0.05 mm) were significantly greater than those in the non-highly myopic group (0.007 ± 0.02 mm) (P < 0.0001). Furthermore, changes in the CNV group (0.081 ± 0.04 mm) were significantly greater than those in the no complications (P < 0.0001) and MTM (0.040 ± 0.05 mm) (P = 0.0059) groups, whereas no significant difference was found between the changes in the MTM and no complications groups (P = 0.91). Multiple regression analyses indicated that CNV eyes (P < 0.0001) and female patients’ eyes (P = 0.04) showed greater changes in the axial length/year.

**Conclusions:**

All groups showed an increase in the axial length, which was greater for highly myopic eyes. In particular, CNV eyes showed greater increases, indicating that larger changes in the axial length may require careful follow-up.

## Introduction

Myopia progresses with an increase in the axial length. When myopia progresses to pathologic myopia, macular complications such as choroidal neovascularization (CNV) and myopic traction maculopathy (MTM) develop and lead to vision loss. The increase in the axial length may be largely associated with clinical characteristics of these complications.

It is considered that the axial length reaches adult length by the age of 13 years [1], showing no further increase in length. Recent cross-sectional studies found that the axial length in older adults tended to be shorter, suggesting that it may decrease with aging [2,3]. However, in a recent longitudinal study by He et al., slight increase of the axial length in adults (mean refractive error, −0.44 ± 2.21 diopters) was reported [4]. Conversely, an increase in the axial length in adults with highly myopic eyes is common and has been previously shown in longitudinal studies [5–7]. However, to the best of our knowledge, there have been no studies directly comparing the increase in the axial length in adults with non-highly myopic eyes or highly myopic eyes. In addition, because previous studies on the axial length in highly myopic eyes have not compared between eyes with and without macular complications [5–7], their influence on the axial length remains unknown. Thus, we investigated how changes in the axial length differ between non-highly myopic and highly myopic eyes with or without complications, particularly CNV and MTM.

## Methods

### Subjects

The database of 1002 eyes of 501 patients seen at the high myopia clinic of Tsukazaki Hospital between April 2010 and March 2016 was studied retrospectively. All patients underwent a complete ophthalmologic examination at each visit, including best-corrected visual acuity using Landolt chart, refractive errors, intraocular pressure, color photography of the fundus, and optical coherence tomography (OCT, DRI OCT-1; Topcon Corp, Tokyo, Japan and/or Heidelberg Spectralis OCT; Heidelberg Engineering, Heidelberg, Germany). The axial length was measured using ocular biometry (IOLMaster; Carl Zeiss Meditec, Jena, Germany) at the first visit, and thereafter every 1 year. The measurements by the IOLMaster were repeated five times at each examination, and the average value was used for the analyses. Inclusion criteria were: (1) age at the enrollment of 30 years or more, (2) highly myopic eyes, axial length of 26 mm or more or myopic refractive error of −6.0 diopters or worse because macular complications are more likely to occur in these eyes, (3) best-corrected visual acuity of 0.6 decimal fraction or better for an accurate measurement of the axial length, and (4) having received several axial length measurements with at least 1 year interval between measurements. Exclusion criteria were as follows: (1) eyes with severe cataract that would prevent accurate measurement of the axial length (grade 3 or more, Emery and Little classification), (2) poor fixation, and (3) history of intraocular surgery, such as cataract surgery in the last 12 months. If the patient underwent an intraocular surgery, such as cataract surgery, during the follow-up period, either preoperative or postoperative measurement was analyzed, and the changes between preoperative and postoperative axial length measurements were not included in the analysis considering the surgical effect on the axial length. In addition, measurements of the axial length were not included in the analysis if exudative changes in eyes with CNV or retinal detachment in eyes with MTM were present at the time of the measurement, even when their visual acuity were good. Additionally, when both eyes had the same ocular condition, one eye was randomly selected and included in the study. This research adhered to the Declaration of Helsinki and was approved by the ethics committee of Tsukazaki Hospital. Since this was a study that retrospectively reviewed medical records and there were no anonymous issues involved, the institutional review board of Tsukazaki Hospital waived the need for consent.

### Classification by myopic macular complications

Of the 1002 eyes from 501 participants, 703 eyes from 501 participants were excluded: 28 eyes from 14 participants because their age was less than 30 years, 42 eyes from 42 participants because their axial length was less than 26.0 mm, 184 eyes from 140 participants because their best-corrected visual acuity was less than 0.6 decimal fraction, 234 eyes from 117 participants because they had not undergone axial length measurement that meets the criteria, 58 eyes from 31 participants because they had a severe cataract or poor fixation, 6 eyes from 6 participants because they had MTM and CNV, and 151 eyes from 151 participants because their fellow eyes had the same ocular condition. Of all eyes (299 eyes from 299 participants) that met the inclusion criteria, 32 eyes of 32 patients had MTM (7 males and 25 females; mean age at the initial examination: 63.8 ± 10.6 years; MTM group) and 34 eyes of 34 patients had CNV (6 males and 28 females; mean age at the initial examination: 63.9 ± 11.1 years, CNV group). MTM was diagnosed using OCT images according to the criteria reported by Panozzo et al [8]. Eyes with complications of CNV were evaluated with OCT, fluorescein angiography, and indocyanine green angiography, and the diagnoses were thus confirmed. From 233 highly myopic eyes from 233 participants without macular complications, age-matched 165 eyes of 165 participants (41 males and 124 females; mean age at the initial examination: 63.8 ± 8.9 years; no complications group) were selected by researchers blinded to the study (TS, KO).

### Non-highly myopic eyes

From the database of the Department of Ophthalmology outpatient clinic of Tsukazaki Hospital, 85 eyes with a spherical equivalent (SE) refractive error between −5.00 and +5.00 diopters of 85 patients who were age-matched similarly (22 males and 63 females; mean age at the initial examination, 63.8 ± 7.6 years; non-highly myopic group) were selected.

### Statistical analysis

Changes in the axial length were determined by paired t-test. The Pearson's chi-square test or Fisher’s exact test were used to analyze categorical variables. The changes in the axial length per year was obtained by dividing the changes in the axial length by the follow-up period. The characteristics or the changes in the axial length per year between the groups were analyzed using the Mann–Whitney U test or the Steel–Dwass test. In addition, we analyzed factors [sex, age at the initial examination, lens condition (phakic or pseudophakic), presence of CNV, axial length at the initial examination, intraocular pressure at the initial examination, and, follow-up period] that might be associated with changes in the axial length per year using stepwise analysis and multiple regression. All statistical analyses were performed using a commercially available software program (JMP, version 10.0, SAS Institute, Inc.). A P value of <.05 was considered to be statistically significant.

## Results

Patient demographic data are shown in Table 1. No significant differences were found between the non-highly myopic group and no complications group with respect to sex, mean age, and mean intraocular pressure at the initial examination. For the mean refractive error at the initial examination, the no complications group showed more severe myopia than the non-highly myopic group (non-highly myopic group, −0.07 ± 2.1 D; no complications group, −13.4 ± 4.2 D; P < 0.0001). Moreover, the axial length at the initial examination was longer in the no complications group than in the non-highly myopic group (non-highly myopic group, 23.6 ± 1.0 mm; no complications group, 29.0 ± 1.6 mm; P < 0.0001) (Table 2). Comparison of the three highly myopic eye groups revealed no significance differences in sex, mean age, mean refractive error, mean axial length, and mean intraocular pressure at the initial examination (Table 3).

**Table 1:** Clinical characteristics at the initial examination.

Characteristic	Non-highly myopic eyes (n = 85)	Highly myopic eyes		
		No complications (n = 165)	Myopic traction maculopathy (n = 32)	Choroidal neovascularization (n = 34)
Sex (number of male/female)	22/63	41/124	7/25	6/28
Age (years) mean ± SD	63.8 ± 7.6	63.8 ± 9.0	63.8 ± 10.6	63.9 ± 11.3
Refractive error (D) mean ± SD	−0.07 ± 2.1	−13.4 ± 4.2	−13.8 ± 4.2	−12.1 ± 3.5
Axial length (mm) mean ± SD	23.6 ± 1.0	29.0 ± 1.6	29.2 ± 1.7	29.5 ± 1.3
Intraocular pressure (mmHg) mean ± SD	14.9 ± 2.8	14.7 ± 2.6	15.1 ± 2.7	15.1 ± 2.3
Follow-up period (months) mean ± SD	33.0 ± 16.9	28.0 ± 17.4	21.6 ± 11.4	28.1 ± 16.2

**Table 2:** Comparison of clinical characteristics between the non-highly myopic eyes and the highly myopic eyes with no complications.

Characteristic	Non-highly myopic eyes (n = 85)	Highly myopic eyes with no complications (n = 165)	P value (Mann-Whitney U test)
Sex (number of male/female)	22/63	41/124	0.86
Age at initial examination (years) mean ± SD	63.8 ± 7.6	63.8 ± 9.0	0.88
Refractive error at initial examination (D) mean ± SD	−0.07 ± 2.1	−13.4 ± 4.2	< 0.0001*
Axial length at initial examination (mm) mean ± SD	23.6 ± 1.0	29.0 ± 1.6	< 0.0001*
Intraocular pressure at initial examination (mmHg) mean ± SD	14.9 ± 2.8	14.7 ± 2.6	0.69

*Statistically significant difference

**Table 3:** Comparison of clinical characteristics between highly myopic eyes with no complications, with myopic traction maculopathy, and with choroidal neovascularization.

Characteristic	Highly myopic eyes			P value (Steel-Dwass test)			P value (Chi-square tests)
	No complications (n = 165)	Myopic traction maculopathy (MTM) (n = 32)	Choroidal neovascularization (CNV) (n = 34)	No complications vs MTM	No complications vs CNV	MTM vs CNV	
Sex (number of male/female)	41/124	7/25	6/28				0.65
Age at initial examination (years) mean ± SD	63.8 ± 9.0	63.8 ± 10.6	63.9 ± 11.3	0.91	0.76	0.97	
Refractive error at initial examination (D) mean ± SD	−13.4 ± 4.2	−13.8 ± 4.2	−12.1 ± 3.5	0.81	0.61	0.53	
Axial length at initial examination (mm) mean ± SD	29.0 ± 1.6	29.2 ± 1.7	29.5 ± 1.3	0.86	0.22	0.83	
Intraocular pressure at initial examination (mmHg) mean ± SD	14.7 ± 2.6	15.1 ± 2.7	15.1 ± 2.3	0.69	0.57	0.97	

The axial length at the initial and final examinations for each group are shown in Table 4. The changes in the axial length for each group were as follows: non-highly myopic group, 0.02 ± 0.06 mm; no complications group, 0.09 ± 0.13 mm; MTM group, 0.08 ± 0.10 mm; and CNV group, 0.19 ± 0.15 mm. We observed a significant increase in all groups (P = 0.008 [non-highly myopic group], P < 0.0001 [no complications group, MTM group, and CNV group]).

**Table 4:** Comparison of axial length (mm) between at the initial examination and at the final examination in each group.

Group	Initial examination	Final examination	Changes of Axial length	P value (Paired t test)
Non-highly myopic group (mm) mean ± SD	23.58 ± 1.0	23.60 ± 1.0	0.02 ± 0.06	0.008*
Highly myopic eyes (mm) mean ± SD				
No complications (n = 165)	29.01 ± 1.6	29.11 ± 1.6	0.09 ± 0.13	< 0.0001*
Myopic traction maculopathy (n = 32)	29.16 ± 1.7	29.24 ± 1.7	0.08 ± 0.10	< 0.0001*
Choroidal neovascularization (n = 34)	29.47 ± 1.3	29.66 ± 1.3	0.19 ± 0.15	< 0.0001*

*Statistically significant difference

Since the mean follow-up period varies in the three groups, it is not possible to directly compare the changes in the axial length. Therefore, we compared the changes in the axial length per year (mm). The changes in the axial length per year were as follows: non-highly myopic group, 0.007 ± 0.02 mm; no complications group, 0.041 ± 0.05 mm; MTM group, 0.040 ± 0.05 mm; and CNV group, 0.081 ± 0.04 mm. The changes in the axial length per year was significantly greater in the no complications group than that in the non-highly myopic group (P < 0.0001) (Table 5). In comparison of the three groups with highly myopic eyes, the CNV group showed the greatest changes in the axial length per year (P < 0.0001 [vs. no complications group], P = 0.0059 [vs. MTM group]), and no significant difference was found between the no complications and MTM groups (P = 0.91) (Table 5).

**Table 5:** Comparison of the changes in the axial length per year between non-highly myopic eyes and highly myopic eyes with no complications and between highly myopic eyes with myopic traction maculopathy, choroidal neovascularization, and no complications.

		Change of Axial length/year (mm) mean ± SD	P value		
			Mann-Whitney U test	Steel-Dwass test	
			vs NC	vs MTM	vs CNV
Non-highly myopic eyes		0.007 ± 0.02	< 0.0001*	−	−
Highly myopic eyes	No complications (NC, n = 165)	0.041 ± 0.05	−	0.91	< 0.0001*
	Myopic traction maculopathy (MTM, n = 32)	0.040 ± 0.05	−	−	0.0059*
	Choroidal neovascularization (CNV, n = 34)	0.081 ± 0.04	−	−	−

*Statistically significant difference

To investigate the factors affecting changes in the axial length per year, the following factors were analyzed for highly myopic eyes using stepwise analysis: sex, age at the initial examination, lens condition (phakic or pseudophakic), presence of CNV, axial length at the initial examination, intraocular pressure at the initial examination, and follow-up period. Multiple regression analyses performed on the two factors, sex and presence of CNV, revealed that the presence of CNV (P < 0.0001) and being female (P = 0.04) contributed to greater changes in the axial length per year (Table 6).

**Table 6:** Multiple regression analysis of the changes in axial length per year by sex and choroidal neovascularization.

Factor	Regression coefficient (95% CI)	Standard error	P value
Intercept	0.056 (0.047–0.066)	0.005	< 0.0001*
Sex (female)	0.008 (0.0004–0.015)	0.004	0.04*
With choroidal neovascularization	0.020 (0.011–0.028)	0.004	< 0.0001*

R2 = 0.10

*Statistically significant difference

## Discussion

In this study, all groups (non-highly myopic, no complications, MTM, and CNV) showed a significant increase in the axial length during the follow-up period. Changes in the axial length per year in the no complications group were significantly greater than those in the non-highly myopic group. Furthermore, changes in the CNV group were significantly greater than those in the no complications and MTM groups. Multiple regression analyses indicated that CNV and female patients’ eyes showed greater changes in the axial length per year. The present study showed a 0.007 mm increase in the axial length of adults with non-highly myopia during the follow-up period. It is considered that the axial length attains the adult length by the age of 13 years and remains the same thereafter [1]. Cross-sectional studies conducted by Gudmundsdottir et al. and Fotedar et al. have revealed the axial length in older adults to be shorter than that in younger adults, suggesting that the axial length decreases with aging [2,3]. However, a recent longitudinal study conducted by He et al. examining the axial length in Chinese adults over a 2-year period using IOLMaster reported a 0.015 mm increase (P < 0.001) [4]. Our study data also supports this result. In the study conducted by He et al., a significant increase in the axial length based on the patients’ age was observed in three of the four subgroups: 45 to 54 years, 0.01 mm (P < 0.01); 55 to 64 years, 0.03 mm (P < 0.001); 65 years or older, and 0.02 mm (P < 0.01). However, in their study, the younger subgroup aged 35 to 44 years showed an increase of 0.004 mm, which was not significant. In highly myopic eyes, a posterior staphyloma begins to form in individuals during their 40s, and the possible causes are the number, the arrangement, and the changes in the type of collagen fibrils in sclera.

A longitudinal study in highly myopic eyes in adults reported an increase in the axial length [5]. In addition, Saka et al. studied the medical records of 184 eyes of 101 high myopia patients with refractive errors of −6 D or more or with axial lengths of 26.5 mm or more and reported a significant increase of the axial length from 28.6 mm to 29.4 mm over the mean follow-up period of 8.2 years [6]. However, A-scan ultrasonography was used in their study. Later, Saka et al., used the IOLMaster, which allows more accurate and non-invasive measurements of the axial length, to examine 185 eyes of 185 high myopia patients (mean age at the initial examination, 48.4 ± 12.2 years) without a history of intraocular surgery and any active fundus diseases, and observed a significant increase in the axial length from 29.35 ± 1.80 mm to 29.48 ± 1.85 mm over a 2-year period [7]. The highly myopic eyes with no complications group in our study also showed an increase of mean axial length from 29.01 ± 1.6 mm to 29.11 ± 1.6 mm, supporting the results of Saka et al. However, their reported changes in the axial length per year was 0.06 mm, which is greater than that found in our study of 0.04 mm per year. The reason for this is not clear; however, their study did not include active CNV but included inactive CNV; therefore, the changes in the axial length per year in their study may have been greater than that in this study. In addition, the differences in patients’ age and axial length at the initial examination may have influenced the results.

In eyes with pathologic myopia, macular complications, such as CNV and MTM, can cause vision loss as aforementioned. The increase in the axial length may be largely associated with the clinical characteristics of these complications. However, to our knowledge, no studies have examined the changes in the axial length in eyes with macular complications. Interestingly, the changes in the axial length per year in the CNV group in our study were significantly greater than those in other groups. Currently, the pathogenesis of myopic CNV (mCNV) is not fully understood. However, it has been reported that lacquer cracks and chorioretinal atrophy can cause mCNV [9,10]. Some studies reported that the axial length was not associated with the pathogenesis of mCNV [9,11]; however, these studies were cross-sectional studies and did not directly investigate the changes in the axial length. Our findings suggest a possible association between the changes in the axial length and mCNV pathogenesis. Measuring the axial length using IOLMaster is easy and non-invasive and can produce accurate measurements. Regular monitoring of patients’ axial lengths is important, and eyes with greater increase should be considered as high risk and may need short-interval follow-up.

Many factors are thought to be involved in the pathogenesis of MTM; in addition to the stretched axial length by a posterior staphyloma, anterior traction and noncompliance of the internal limiting membrane (ILM) are considered to cause MTM [12–14]. In our study, no significant difference was found for the increase in the axial length between the no complications and MTM groups. For this reason, traction or ILM noncompliance may be largely associated with MTM instead of the stretched axial length by a posterior staphyloma.

Miyake et al. and Wakazono et al. analyzed the shape of posterior poles of eyes with and without MTM in high myopia and reported that eyes with MTM were steeper [15,16]. In our study, no difference was observed for the increase in the axial length between the no complications and MTM groups. This finding suggests that although eyes with MTM exhibit changes in the axial length similar to normal myopic eyes, age-related alterations in sclera can contribute to the differences in eye shape. When posterior poles of eyes become steep, anterior retinal traction forces may increase, and as a result, retinal detachment may occur.

Multiple regression analyses in all highly myopic eyes showed that CNV and sex influence the changes in the axial length per year. To note, the presence of CNV and being female contributed to greater changes in the axial length per year. The study by Saka et al., in which changes in the axial length were examined using the IOLMaster, similar to our study, reported that a longer axial length at the initial examination resulted to a greater changes in the axial length per year [7]. However, in our stepwise analysis, the axial length at the initial examination was not selected. This may be due to the difference in items studied between the present study and the study by Saka et al. In general, diseases with collagen abnormalities are more common in females; this may be one of the reasons why the female sex showed greater changes in the axial length per year.

This study had several limitations. First, it was a retrospective study, and it did not show a causal relationship between increase in the axial length and myopic macular complications. Since a previous study suggests that changes in the axial length per year was significantly greater in older patients than in the younger cohort [6], age might have been an important factor; therefore, the subjects were matched for age. Random sampling was performed by blinded researchers; however, sample selection bias may have been present. Height is associated with axial length but was not measured in this study. Since the mean follow-up period was relative short, changes in the axial length may have been smaller those observed in previous studies. In addition, since the mean follow-up period varies in the three groups, we compared the changes in the axial length per year. However, there is a possibility that the changes in the axial length per year may change because of the difference in the follow-up period. Because good visual acuity was a minimum requirement to obtain accurate measurements, severe cases were not included in the study. For example, there might have been some differences in measurements within the CNV group, or changes might have been different in eyes with chorioretinal atrophy. Moreover, we did not examine the data by classifying posterior staphylomas, which may be involved in the development of macular complications. However, determining the presence of a posterior staphyloma is variable and also requires clinical experience. It will be key to find an effective way to monitor and predict the risk of developing mCNV using an easy and non-invasive examination. In the future, we believe that it is important to conduct a prospective study that examines the changes in the axial length in highly myopic eyes with various complications in a large population and systematically identify associations.

## Supporting information

S1 Data Data analyzed. (XLSX)
